# Advances in the study of ELABELA in renal physiological functions and related diseases

**DOI:** 10.3389/fphar.2023.1276488

**Published:** 2023-10-31

**Authors:** YuRong Liu, MingChun Jiang, Yue Li, Peng Chen, XiaoYu Chen

**Affiliations:** ^1^ Department of Physiology and Neurobiology, Shandong First Medical University (Shandong Academy of Medical Sciences), Taian, Shandong, China; ^2^ Department of Anatomy, Shandong First Medical University (Shandong Academy of Medical Sciences), Taian, Shandong, China

**Keywords:** ELABELA, apelin, APJ, acute kidney injury, hypertensive kidney damage, diabetic nephropathy, renal cancer

## Abstract

ELABELA (ELA), also known as Toddler or Apela, is a novel endogenous ligand of the angiotensin receptor AT1-related receptor protein (APJ). ELA is highly expressed in human embryonic, cardiac, and renal tissues and involves various biological functions, such as embryonic development, blood circulation regulation, and maintaining body fluid homeostasis. ELA is also closely related to the occurrence and development of acute kidney injury, hypertensive kidney damage, diabetic nephropathy, renal tumors, and other diseases. Understanding the physiological role of ELA and its mechanism of action in kidney-related diseases would provide new targets and directions for the clinical treatment of kidney diseases.

## 1 Introduction

In 1993, O'Dowd et al. first identified the APJ receptor (putative receptor protein related to the angiotensin receptor AT1) from the human genome (O'Dowd et al., 1993). It is a G protein-coupled receptor (GPCR) with a seven α-transmembrane helices and is also known as an orphan G protein-coupled receptor (oGPCRs) because no endogenous ligands have been identified. The gene sequences of APJ and AT1 receptors share about 35% homology but do not bind to angiotensin II (Read et al., 2019).In 1998, Tatemoto et al. extracted and purified a new neurocardiovascular active peptide, Apelin, from bovine gastric secretions using a reverse pharmacological approach and established it as an endogenous ligand for the APJ receptor (Tatemoto et al., 1998). Apelin and its receptor are distributed in various tissues and organs of the body and are involved in the regulation of cardiovascular activity, angiogenesis, and the adipose islet axis, and play a critical role in the maintenance of body fluid homeostasis (Galanth et al., 2012; Chapman et al., 2014).

In 2013, Chng et al., for the first time, identified another novel endogenous ligand of APJ, ELABELA (ELA), in zebrafish embryos (Chng et al., 2013); also, Pauli et al. reported the same peptide structure and named it Toddler (Pauli et al., 2015). ELA is highly expressed in human embryonic, heart, and kidney tissues, and its role in promoting embryonic development, regulating blood circulation, and maintaining fluid homeostasis is being discovered gradually (Deng et al., 2015; Freyer et al., 2017; Sato et al., 2017). A current study showed that ELA is closely related to the pathophysiological function of the kidney and exerts a protective role in various kidney diseases (Chen et al., 2020a). This article reviews the structure, physiological function, and role of ELA in the development of kidney diseases.

## 2 General biological characteristics of ELA

**FIGURE 1:**
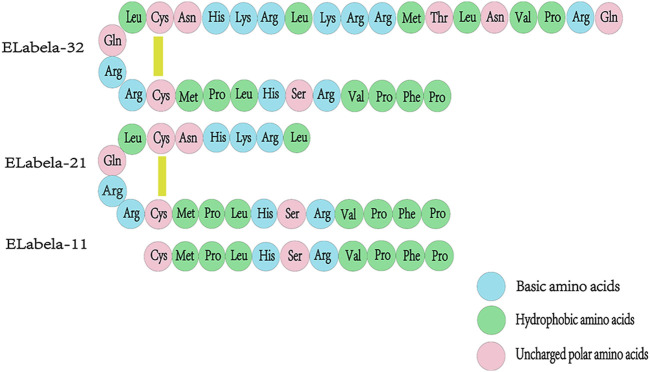
Amino acid sequences of ELA-32, ELA-21, and ELA-11 isoforms. The signal sequence of ELA, a precursor protein consisting of 54 amino acids, is cleaved to produce a mature 32 amino acid peptide (ELA-32). ELA-32 contains two cleavage sites that are cleaved to produce two additional amino acid fragments, ELA-21 and ELA-11, where in the N-terminal Gln of ELA-32 will be converted to pyroglutamic acid. ELA- 32 and ELA-21 form a bridge between Cys residues.

The *ELA* gene of zebrafish is located on chromosome 1 and consists of three exons. ELA is expressed during zebrafish embryogenesis, from the middle blastocyst to 3 days after fertilization (Pauli et al., 2014). The human gene encoding ELA, *AK092578*, is located on chromosome 4 and contains three exons and a non-coding RNA transcript gene (Xie et al., 2014).The ELA mRNA consists a conserved open reading frame (ORF) and encodes a precursor protein composed of 54 amino acids. The N-terminal signal sequence of ELA consists 22 amino acid residues and a mature peptide ELA-32 comprising 22 amino acid residues, of which 13 amino acids at the C-terminal are conserved across vertebrates Under the action of endoplasmic reticulum and Golgi apparatus, ELA-32 is then disassembled into small molecular isoforms, such as ELA-21 and ELA-11(Ma et al., 2021). The specific amino acid sequences of the different isoforms are illustrated in Figure 1.

ELA was first identified in zebrafish embryogenesis, highly expressed in the neural tube (Stock et al., 2022). It mediates endodermal differentiation and cell migration, thereby inducing angiogenesis to promote early cardiac development during zebrafish embryogenesis (Pauli et al., 2014). Thus, ELA plays a critical role in the development of the embryonic cardiovascular system (Eberlé et al., 2019). Human embryonic stem cells (hESCs) also express and secrete ELA. Although ELA maintains self-renewal capacity in hESCs in a paracrine manner, its expression is rapidly downregulated during hESC differentiation (Miura et al., 2004; Ho et al., 2015). In addition to expression during embryonic development, ELA was highly expressed in adult kidney and prostate tissues (Wang et al., 2015; Coquerel et al., 2017; Xie et al., 2022; Xiong et al., 2023). In human vasculature, ELA is more highly expressed in the arteries than in veins but at low levels in the human heart and lung tissues (Yang et al., 2017a).

**FIGURE 2:**
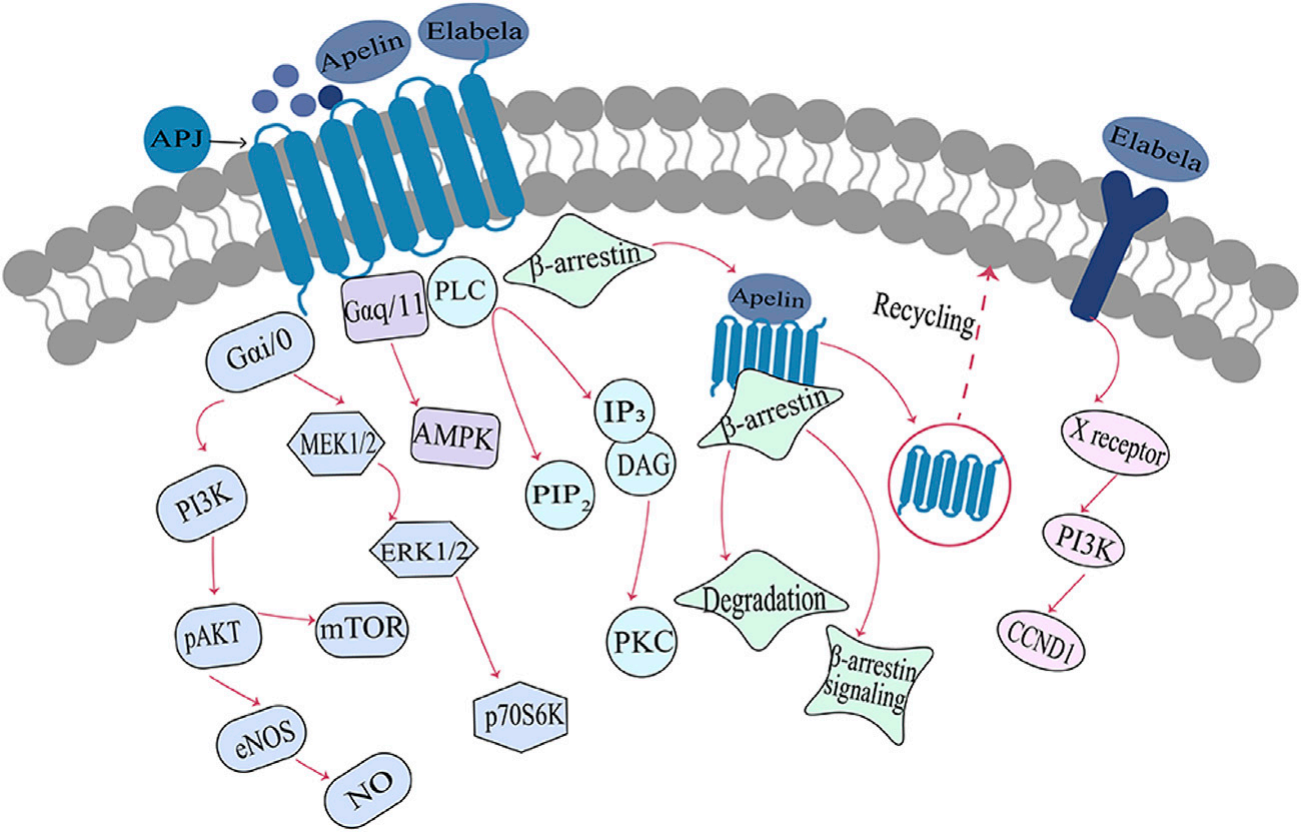
Signaling pathways associated with the Elabela-Apelin-APJ systemNote. After binding with the APJ receptor, ELA/Apelin exerts different biological effects through G-protein-dependent and/or β-arrestin-dependent signaling pathways. ELA, Elabela; X receptor, unknown receptor; PI3K, phosphatidylinositol 3-kinase; pAKT, phosphorylated serine/threonine protein kinase; eNOS, endothelial nitric oxide synthase; NO, nitric oxide; mTOR, rapamycin target protein; MEK1/2, mitogen-activated protein kinase; ERK1/2, extracellular regulatory protein kinase; p70S6, p70 ribosomal S6 kinase; AMPK, AMP-dependent protein kinase; PIP2, phosphatidylinositol diphosphate; IP3, inositol triphosphate; PLC, phospholipase C; PKC, protein kinase C; DAG, diacylglycerol; CCND1, cell cycle protein.

ELA and apelin also act as endogenous ligands for APJ receptors, but their sequence similarity is minimal (Murza et al., 2016). Various isoforms of ELA can bind to APJ; ELA-32 and ELA-21 bind to APJ with subnanomolar and nanomolar affinities significantly better than that of the short peptide ELA-11 interaction with APJ (Yang et al., 2017a). The experiments of alanine scanning and mutational analysis suggest that ELA and apelin bind to different residues in the APJ receptor (Couvineau et al., 2020), resulting in distinct binding patterns between these endogenous ligands and the APJ receptor (Shin et al., 2017). Apelin or ELA binding to APJ initiates intracellular signaling by coupling different G proteins and exerting a wide range of biological effects (Masri et al., 2006; Zhang et al., 2017). On one hand, APJ mediates the vasodilation response of blood vessels through the coupling of Gαi/o to activate downstream ERK and PI3K-AKT signaling cascades (Zhang et al., 2017). On the other hand, APJ activates phospholipase C (PLC) and AMPK signaling by coupling Gαq/11 to enhance myocardial contractility and glucose uptake in skeletal muscle cells (Szokodi et al., 2002; Dray et al., 2008; Murza et al., 2016). Subsequently, the activated APJ receptor is uncoupled from G proteins, following which it mediates receptor endocytosis by recruiting β-arrestin and initiating β-arrestin-dependent intracellular signaling pathways that are involved in a key physiological regulatory processes in the body, including transcription, cell division, and apoptosis (Ceraudo et al., 2014; Ma et al., 2021). In addition to that, ELA can also bind to an unknown receptor and plays a crucial role in cell growth, survival, and self-renewal by activating the PI3K/AKT phosphorylation signaling pathway or inhibiting the p53 signaling pathway(Ho et al., 2017; Sato et al., 2017). (Figure 2)

Compared to [Pyr1]apelin-13, longer ligand isoforms such as apelin-17, apelin-36, ELA-21, and ELA-32 demonstrate a stronger bias towards the β-arrestin signaling pathway (Yang et al., 2017a; Yang et al., 2017b; Nyimanu et al., 2019). Our team’s previous research has also confirmed that apelin and ELA form a spatiotemporal dual-ligand system, exhibiting distinct signaling profiles upon binding to the APJ receptor (Nyimanu et al., 2019). ELA-32 and apelin-17 display a stronger preference for β-arrestin-dependent signaling pathways, while ELA-21 and pGlu1-apelin-13 exhibit more pronounced activity in G-protein-dependent signaling pathways (Chen J. et al., 2020). The involvement of this bias in the physiological functions and pathophysiology of APJ requires further investigation. In conclusion, the ligand activity and signaling bias demonstrated by different apelin and ELA isoforms contribute to a better understanding of the functionality of the APJ receptor and provide more options for drug development targeting this receptor.

## 3 Physiological role of ELA in the kidney

Apelin and its receptor APJ are widely distributed in various tissues and highly expressed in glomerular and tubular structures in humans and rats (Hosoya et al., 2000). While ELA is mainly distributed in the kidney, RNAscope and immunofluorescence detected that these are concentrated on the apical membrane of the main cells of the outer to inner medullary collecting ducts in rats and co-localized with the AQP-2 marker of the main cells (Xu C. et al., 2020). A previous study showed that either lateral ventricular or intravenous administration of apelin or ELA significantly increases the urine output of the animals (Deng et al., 2015; Jiang et al., 2021). These findings suggested that ELA may directly affect the kidney, regulating renal hemodynamics and the substance transport function of the renal tubules and collecting ducts.

**FIGURE 3:**
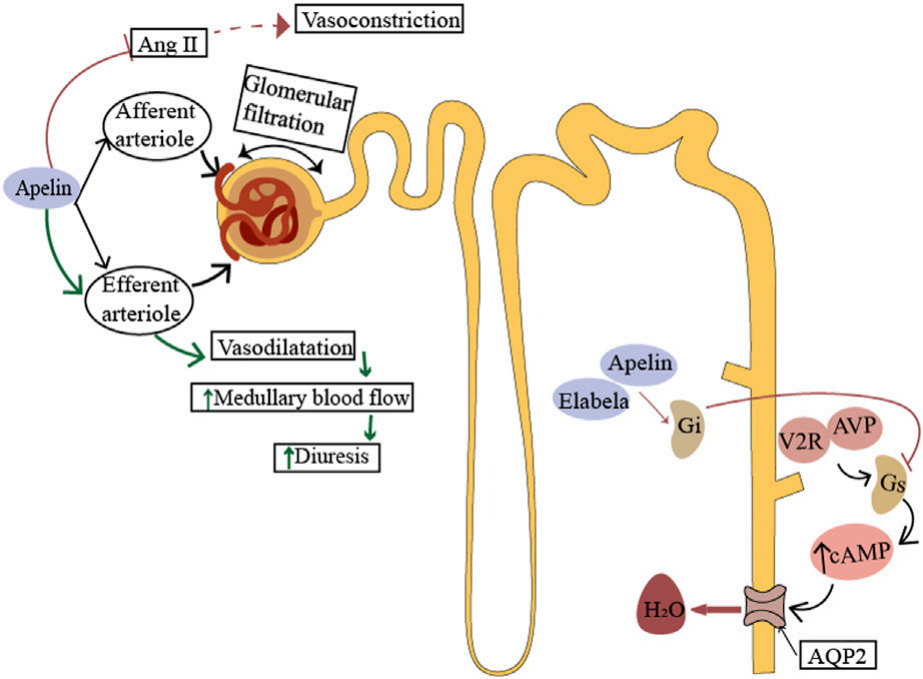
Physiological role of ELA in the kidney. Apelin acts on the afferent and efferent arterioles, promotes vasodilation by producing nitric oxide (NO), and counteracts the effect of Ang II. The action of apelin antagonizes vasopressin signaling in renal tubules. In the principal cells of the collecting duct, apelin or elabela prevents pressor-induced translocation of aquaporin 2 (AQP2) to the apical membrane, thereby preventing water reabsorption. Afferent arterioles, small inlet arterioles; efferent arteriole, small outlet arterioles; AT1 receptor, angiotensin II-1 type 1 receptor; AVP, arginine vasopressin; V2R, arginine vasopressin type II receptor; AQP2, aquaporin 2.

Renal blood flow is tightly regulated by renin-angiotensin system (RAS), which in turn affects glomerular blood flow and glomerular filtration rate (Gerbier et al., 2017). Apelin inhibits angiotensin II-induced contractile activity of the small glomerular inlet and outlet arteries (Hus-Citharel et al., 2008), regulates renal hemodynamics, and increases renal blood flow. This might be one of the mechanisms by which apelin produces its diuretic effect, but it is dependent on intact vascular endothelium and nitric oxide (NO) production (Hus-Citharel et al., 2008; Wang et al., 2015; Yang et al., 2015). On the other hand, since the straight small vessels receive blood supply from the small outflowing arteries, the apelin-induced vasodilatory response may promote diuresis by increasing renal medullary blood flow. Studies have demonstrated that ELA can also counteract the action of the renin-angiotensin system (RAS) in the distal renal tubules, exerting physiological effects similar to apelin (Chapman et al., 2021). (Figure 3)

In addition, APJ expression was progressively increased along the rat cortex to the inner medullary collecting duct (Hus-Citharel et al., 2014). Apelin acts directly on the renal tubules, exerting a diuretic effect in a dose-dependent manner and reducing urine osmolality (Hus-Citharel et al., 2014; 2008). *In vivo* and *in vitro* studies have shown that apelin affects water reabsorption and regulates urine output by inhibiting the translocation of water channel protein-2 to the apical membrane (Boulkeroua et al., 2019). In rodent kidneys, ELA is more abundantly expressed than apelin and is predominant in the medullary collecting ducts (O'Carroll et al., 2017). In adult rats, urinary flow rate and water uptake were significantly increased after intravenous administration of ELA or apelin. Interestingly, the effect of ELA was five times greater than that of apelin, suggesting that ELA is involved in the regulation of renal function (Deng et al., 2015). The urine flow rate and water intake in rats were inhibited by the use of ELA antagonist (ELA-PA). In addition, under pathological conditions of hypertension, long-term use of ELA can reduce the excretion of electrolyte sodium and chlorine, possibly through the regulation of osmotic pressure through reabsorption of sodium chloride (Schreiber et al., 2017). Since ELA expression is confined to the vascular endothelium and kidney in adults, it may play a critical role in the regulation of fluid homeostasis.

The specific mechanism by which ELA maintains fluid balance is not yet fully elucidated. In the central nervous system, apelin and arginine vasopressin (AVP) coexist in the same hypothalamic neuroendocrine neurons (Reaux et al., 2001; De Mota et al., 2004). Apelin, as a potent neurodiuretic peptide, directly inhibits the periodic firing activity of AVP neurons and the release of AVP through the APJ receptor, antagonizing the effects of AVP (Reaux-Le Goazigo et al., 2004; Azizi et al., 2008). Studies have shown that neuroendocrine neurons in the hypothalamus of humans and other mammals regulate the secretion of apelin and AVP in opposite directions via a “Yin-Yang” pattern in response to stimuli of different osmotic pressures. This control of AVP release and antidiuretic effect at the “optimal level” is crucial for maintaining body fluid balance (Girault-Sotias et al., 2021). It has been found that intracerebroventricular injection of ELA in mice can activate AVP neurons in the paraventricular nucleus (PVN), reduce food intake, and exert anorectic effects (Girault-Sotias et al., 2021). Microinjection of ELA-21 into the PVN increased renal sympathetic nerve activity and AVP levels in spontaneously hypertensive rats (Geng et al., 2020). However, it remains to be further studied whether ELA and AVP also maintain fluid balance through opposing regulatory effects.

## 4 Role of ELA in kidney disease

Recent studies have demonstrated a protective role of ELA in kidney-related diseases. ELA treatment preserves glomerular structure, inhibits the expression of pro-fibrosis-related genes in rat kidneys, and improves renal fibrosis in rats (Schreiber et al., 2017; Xu C. et al., 2020; Chen et al., 2020c). When ELA levels in serum were reduced, renal function was impaired and the progression of renal disease was accelerated (Lu et al., 2020). These findings suggested that ELA is involved in the pathophysiological process of renal disease development and is a novel therapeutic target for renal disease.

### 4.1 ELA and acute kidney injury

Acute kidney injury, mainly caused by ischemia/reperfusion injury (IRI) and nephrotoxins, is characterized by rapid deterioration of renal function and high mortality; however, there is a lack of effective clinical treatment(Sharma et al., 2019). The pathological process of IRI includes inflammation and apoptosis(Bonventre and Zuk, 2004; Wang et al., 2020). *In vitro* studies found that the overexpression of ELA-32 and ELA-11 in renal tubular epithelial cells (NRK-52E) significantly inhibits cellular DNA damage, apoptosis, and inflammatory response induced by I/R or adriamycin treatment (Chen et al., 2017). Further *in vivo* experiments revealed that ELA expression levels were significantly reduced in mouse kidneys in the renal I/R injury model. The expression levels of inflammatory factors (IL-6, IL-8, and MCP1), kidney injury factor (KIM-1), fibrotic factors (vimentin, TGF-β1, fibronectin, collagen1a), DNA damage markers, and apoptotic factors were significantly elevated. At the same time, ELA-32 and ELA-11 inhibited I/R injury-induced renal fibrosis, inflammation, apoptosis, DNA damage response, and macrophage infiltration and attenuated tubular lesions and pathological scores of renal function (Chen et al., 2015; Chen et al., 2017). Furthermore, ELA improved the levels of blood creatinine, 24-h urine volume, proteinuria, urinary creatinine, and urea nitrogen in mice. Compared to ELA-32, ELA-11 showed better protection against I/R injury-induced DNA damage response, apoptosis, and cell viability (Chen et al., 2017). In addition, only ELA-11 showed significant inhibition of renal I/R injury-induced autophagy, although the mechanism of action is unclear(Chen et al., 2017). To further clarify whether ELA is involved in the protective effect against renal I/R injury through APJ receptor, Chen et al. knocked down APJ using siRNA in I/R-induced NRK-52E cells. This significantly enhanced cell viability and inhibited inflammatory responses and DNA damage effects, suggesting that the protective role of ELA in I/R is independent of the presence of APJ, and that ELA exerts nephroprotective effects through other unknown receptors (Chen et al., 2017).

Acute kidney injury caused by sepsis is a common clinical condition, and ELA has been reported to ameliorate cardiorenal injury in sepsis (Singbartl and Kellum, 2012). ELA is a small molecule peptide with short half-life *in vivo*, rendering it unsuitable for clinical treatment. Xu et al. fused the Fc structural domain of human immunoglobulin IgG with ELA-21 to produce Fc-ELA, which prolonged the plasma half-life of ELA and made it biologically active(Xu F. et al., 2020). In a mouse model of liposaccharide (LPS)-induced acute kidney injury, Fc-ELA fusion protein significantly ameliorated LPS-induced kidney injury and attenuated macrophage infiltration, renal inflammation, and apoptotic response(Wang, 2020). Therefore, Fc-ELA fusion protein has a significant nephroprotective effect on LPS-induced acute kidney injury and could be used in the treatment of the disease.

### 4.2 ELA and hypertensive kidney damage

Hypertensive renal damage is the structural and functional damage to the kidney caused by primary hypertension, which has become the third cause of end-stage renal disease (ESRD) after primary glomerular disease and diabetic nephropathy (Bidani and Griffin, 2004). Hypertension-triggered RAS is involved in the pathophysiology of hypertensive renal damage (Yang and Xu, 2017; Xu et al., 2021). Sato et al. found that exogenous ELA inhibited Ang Ⅱ-induced hypertension in C57/BL6J wild-type mice and significantly reduced Ang Ⅱ-induced myocardial fibrosis, suggesting that ELA antagonizes the RAS (Sato et al., 2017). In high salt-loaded Dahl salt-sensitive rats, exogenous administration of ELA-32 significantly reduced blood pressure and proteinuria levels and was accompanied by a decline in the levels of soluble (pro) renin receptor (sPRR), Angiotensin converting enzyme (ACE), Ang Ⅱ, and reninogen/renin expression in the renal medulla of rats (Xu C. et al., 2020). ELA also significantly reduced the expression of inflammatory factors, renal fibrosis markers, and renal injury markers in high salt-induced hypertensive rats, confirming its role in regulating blood pressure and ameliorating hypertensive renal damage by antagonizing intrarenal RAS (Xu C. et al., 2020).

Schreiber et al. mediated *ELA* gene expression in Dah1 salt-sensitive rats by injection of adeno-associated virus type 9 (AAV9) vector. The study also used a high-salt diet to establish a hypertension rat model and found that ELA delayed the development of elevated blood pressure in animals (Schreiber et al., 2017). After 12 weeks of ELA treatment, the excretion of sodium and chloride gradually decreased in rats, glomerulosclerosis and tubular injury were reduced, and the transcript levels of transforming growth factor β (TGF-β), type 1 collagen and fibronectin, and marker genes of renal fibrosis were decreased, suggesting that the sustained expression of ELA significantly reduces the blood pressure. Also, inhibition of the gene expression of fibrotic factors decreases interstitial fibrosis in the kidney(Schreiber et al., 2017). Thus, ELA may improve renal damage caused by long-term hypertension by inhibiting the activity of RAS and the development of interstitial fibrosis. These findings indicate that ELA provides a potential long-term treatment for hypertension and renal remodeling.

### 4.3 ELA and diabetic nephropathy

Diabetic kidney disease (DKD) is one of the most common and serious chronic microvascular complications of diabetes mellitus. It has become a major cause of ESRD and is considered an independent risk factor for many cardiovascular diseases (Umanath and Lewis, 2018; Zheng et al., 2018). DKD is clinically characterized by increased urinary protein excretion and loss of renal function, manifested by glomerular hypertrophy, hypofiltration, and renal fibrosis (Oshima et al., 2021). Clinical studies have found that serum ELA levels are reduced in patients with DKD and are significantly negatively correlated with urinary albumin/creatinine ratio (ACR) and serum creatinine, suggesting that the molecules is involved in the progression of DKD (Zhang et al., 2018).Therefore, the decrease in serum ELA levels may be a clinical predictive indicator for DKD patients, and may also become a new therapeutic drug for preventing or delaying the progression of DKD. However, currently we need to conduct more clinical studies to evaluate its safety and effectiveness in greater depth.

As a key regulator of diabetic glomerular injury, damage and a reduced number of podocytes are closely associated with the production of DKD proteinuria (Guo et al., 2015). In an STZ-induced type 1 diabetic mouse model, Zhang et al. observed decreased expression of the podocyte-specific associated proteins, synaptopodin and podocin (Zhang et al., 2018). ELA upregulates synaptopodin and podocin expression through PI3K/AKT/mTOR signaling pathway and reduces the apoptosis of foot cells (Zhang et al., 2019). In addition, ELA treatment for 6 months inhibited the renal inflammatory and fibrotic responses in diabetic mice and significantly improved renal dysfunction, thus exhibiting a protective effect against diabetic nephropathy.

Oxidative stress leads to excessive accumulation of reactive oxygen species (ROS) and causes kidney damage, which is crucial in the pathogenesis of DKD. Xu et al. confirmed that ELA reduces LPS-induced ROS production in kidney and renal tubular epithelial cells, prevents cell apoptosis, and participates in ELA-mediated cell protection through the PI3K/AKT signaling pathway(Xu F. et al., 2020). Furthermore, overexpression of renal ELA blocked the NADPH oxidase/ROS/NLRP3 inflammasome pathway and reduced ROS production in DOCA/salt-induced hypertensive rat kidneys. Thus, it could be deduced that ELA improves oxidative stress during DKD by inhibiting this pathway and exerts a protective effect on the kidneys (Chen et al., 2020a).

### 4.4 ELA and kidney cancer

As one of the most common malignant tumors worldwide, kidney cancer has become a critical disease that threatens human health and life safety (Motzer et al., 1996; Curti, 2004). ELA promotes tumor cell proliferation, reduces apoptosis, and is closely related to tumor growth (Acik et al., 2019). A previous study examined ELA’s immunoreactivity in different Fuhrman renal cell carcinoma grades. No immunoreactivity to ELA was detected in Fuhrman grade 1 and 2 clear cell renal cell carcinoma, while that in Fuhrman grade 3 and 4 clear cell renal cell carcinoma was significantly lower than that in normal kidney tissue and that in grade 4 clear cell renal cell carcinoma was significantly higher than that in grade 1, 2, and 3 (Artas et al., 2019). Also, differences in ELA immunoreactivity were observed while comparing benign eosinophiloma and renal chromocytocarcinoma, and ELA expression in renal chromocytoma was significantly higher than in benign eosinophiloma of the kidney (Artas et al., 2019). This phenomenon suggested that ELA is critical in the differential diagnosis of renal tumor pathology.

The Cancer Genome Atlas (TCGA) data showed that the expression of *ELA* gene (*APELA*) was upregulated in the colon, lung, gastric, and thymoma cancers; however, *APELA* was systematically downregulated in all kidney cancer types, including renal chromocytocarcinoma, papillary kidney cancer, and clear cell renal cell carcinoma.Moreover, APELA is negatively correlated with the expression of the cell proliferation marker Ki-67 in the above kidney cancer. These data suggested the role of *APELA* in human kidney cancer and its negative association with the development of kidney cancer (Artas et al., 2019; Linehan and Ricketts, 2019; Liu et al., 2021). In the *in vivo* animal experiments, subcutaneous injection of ELA into kidney cancer model mice decreased the tumor growth in mice bearing ELA tumors (Soulet et al., 2020; Liet et al., 2021) ELA-11, ELA-32, and mut ELA-32 induced mTORC1 activation, inhibited ERK and AKT activation, and promoted apoptosis of renal cancer cells to exert anti-tumor effects. However, the combination of mut ELA and the angiogenesis inhibitor sunitinib (Sutent) enhances the effect of ELA in inhibiting tumor growth (Soulet et al., 2020). The replacement of arginine with serine residues in the protopeptide ELA-32 blocked the conversion of ELA32 to ELA11, allowing ELA-32 to mediate APJ endocytosis and recirculation rapidly, providing it a high affinity for APJ, facilitating better antitumor effects than the less stable protopeptide ELA (Soulet et al., 2020). Therefore, ELA or its derivatives have potential applications in the treatment of kidney cancer.

## 5 Discussion and outlook

**FIGURE 4:**
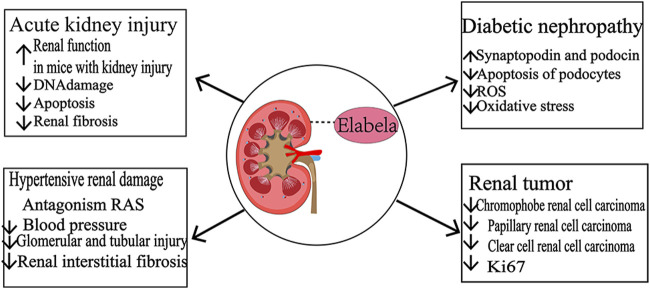
Role of ELA in kidney-related diseases.Acute kidney injury. ELA improves kidney function and attenuates DNA damage, apoptosis, and renal fibrosis in mice with kidney injury. Hypertensive kidney damage: ELA antagonizes the effect of RAS, reduces blood pressure, and attenuates glomerular, tubular injury, and interstitial fibrosis. Diabetic nephropathy: upregulation of synaptopodin and podocin expression decreases the death of podocytes, reduces LPS-induced ROS production in kidney and tubular epithelial cells, improves oxidative stress, and exerts renal protective effects. Renal tumors: ELA was systematically downregulated in renal suspensory cell carcinoma, papillary renal carcinoma, and clear cell renal cell carcinoma, and ELA was negatively correlated with the expression of Ki-67, a cell proliferation marker.

Apelin and its receptor APJ are widely distributed in the body and are involved in various physiological functions, including cardiovascular function regulation, neuroendocrinology, glucose metabolism, and angiogenesis. ELA is a novel endogenous agonist of the Apelin receptor with a similar role. In addition to its critical role in embryonic cardiovascular development, ELA is highly expressed in the adult kidney, where it is involved in renal excretory functions and maintains body fluid homeostasis. In addition, ELA is closely associated with the development of various renal diseases, including acute kidney injury, hypertensive kidney damage, diabetic nephropathy, and renal tumors (Figure 4), thereby deeming it a promising biomarker to identify different types of renal tumors. The discovery and exploration of ELA has been expanded in the pathogenesis of renal diseases, providing new targets and directions for treating related diseases. Several studies have found that the protective effects of ELA in kidney-related diseases are independent of APJ occurrence. The other receptors that bind to and act on ELA are yet to be identified, and the specific signaling mechanisms are to be elucidated. The current studies on ELA in kidney-related diseases are mainly focused on the cellular, molecular level and animal models, lacking large-scale clinical trials that need to be conducted in the future.
